# Proline metabolism is essential for alkaline adaptation of Nile tilapia (*Oreochromis niloticus*)

**DOI:** 10.1186/s40104-024-01100-w

**Published:** 2024-10-14

**Authors:** Minxu Wang, Yuxi Yan, Wei Liu, Jinquan Fan, Erchao Li, Liqiao Chen, Xiaodan Wang

**Affiliations:** Laboratory of Aquaculture Nutrition and Environmental Health, School of Life Sciences, East China Normal University, Shanghai, 200241 China

**Keywords:** Alkalinity stress, *Oreochromis niloticus*, Oxidative stress, Proline metabolism, Pyrroline-5-carboxylate reductase

## Abstract

**Background:**

Saline-alkaline water aquaculture has become a key way to mitigate the reduction of freshwater aquaculture space and meet the increasing global demand for aquatic products. To enhance the comprehensive utilization capability of saline-alkaline water, it is necessary to understand the regulatory mechanisms of aquatic animals coping with saline-alkaline water. In this study, our objective was to elucidate the function of proline metabolism in the alkaline adaptation of Nile tilapia (*Oreochromis niloticus*).

**Results:**

Expose Nile tilapia to alkaline water of different alkalinity for 2 weeks to observe changes in its growth performance and proline metabolism. Meanwhile, to further clarify the role of proline metabolism, RNA interference experiments were conducted to disrupt the normal operation of proline metabolic axis by knocking down *pycr* (pyrroline-5-carboxylate reductases), the final rate-limiting enzyme in proline synthesis. The results showed that both the synthesis and degradation of proline were enhanced under carbonate alkalinity stress, and the environmental alkalinity impaired the growth performance of tilapia, and the higher the alkalinity, the greater the impairment. Moreover, environmental alkalinity caused oxidative stress in tilapia, enhanced ion transport, ammonia metabolism, and altered the intensity and form of energy metabolism in tilapia. When the expression level of the *pycr* gene decreased, the proline metabolism could not operate normally, and the ion transport, antioxidant defense system, and energy metabolism were severely damaged, ultimately leading to liver damage and a decreased survival rate of tilapia under alkalinity stress.

**Conclusions:**

The results indicated that proline metabolism plays an important role in the alkaline adaptation of Nile tilapia and is a key regulatory process in various biochemical and physiological processes.

## Introduction

In recent years, global climate change and human activities have resulted in a worldwide decline in fresh water resources, directly reducing the available space for freshwater aquaculture and limiting its development. Therefore, exploring alternative aquaculture water types is beneficial for promoting global aquaculture development [1–3]. Saline-alkaline water is distributed in over 100 countries, and although it characterized by high salinity, alkalinity, and pH levels, it still has great potential and application value [4, 5]. Previous reports indicated that certain economically species can be raised in inland alkaline brackish water, such as pacific white shrimp (*Litopenaeus vannamei*), Lake Qinghai scaleless carp (*Gymnocypris przewalskii*), blue mussel (*Mytilus edulis*), razor clam (*Sinonovacula constricta*), and Nile tilapia (*Oreochromis niloticus*) [6–10]. Although these species can survive in certain saline-alkaline conditions, their growth performance, alkalinity tolerance, and other aspects are still affected. Therefore, understanding the saline-alkaline tolerance regulation mechanisms of saline-alkaline tolerant species remains an important issue for promoting the development of saline-alkaline water aquaculture.

High carbonate alkalinity is considered to be one of the main risk factor for aquatic animals in saline-alkaline water [11]. Studies have shown that as the salinity and alkalinity of water increase, the toxic effects of CO_3_^2−^ and HCO_3_^−^ on aquatic animals also increase. This negatively affects the growth and development, tissue structure, respiratory system, nutritional metabolism, antioxidant defense system, immune regulation, and intestinal health of aquatic animals, leading to various abnormal physiological and biochemical manifestations and even mortality [12, 13]. Further studies have shown that amino acid metabolism and lipid metabolism were affected by saline-alkaline environment, including glutamine metabolism, which has been reported in fish [2, 14, 15]. However, the process and regulatory mechanisms of adaptation of aquatic animals to saline-alkaline environments remain incompletely understood.

It has been proved that the accumulation of proline occurred under stress condition in various organisms [16, 17]. As a proteinogenic amino acid with special conformational rigidity, proline has multiple functions, playing various roles in osmoprotection, antioxidant reactions, protein synthesis, cell signal transduction, apoptosis, and cell survival [18, 19]. The accumulation of proline is regulated by the balance between biosynthesis and degradation, forming a cycle with glutamate and ornithine as the main sources of proline biosynthesis and its final product of catabolism [18]. The entire process involves multiple metabolic enzymes, among which pyrroline-5-carboxylate reductases (PYCRs) are the final enzyme in proline synthesis, and proline dehydrogenase (PRODH) is the first enzyme in proline degradation [20]. Proline metabolism plays a crucial role in regulating intracellular redox homeostasis, participating in energy storage and transfer, energy reduction, and coupling with the mitochondrial respiration through the tricarboxylic acid cycle (TCA) and the electron transport chain (ETC), thereby affecting cell survival and death [21, 22]. Therefore, compared with the characteristics of the amino acid itself, the dynamic changes of proline metabolism may be more conducive to enhance the tolerance of organisms under environmental stress [23]. Under alkaline conditions, organisms undergo a series of changes in amino acid metabolism and content to maintain homeostasis [24–27]. However, whether proline metabolism plays an important role in the alkaline adaptation in fish is largely unknown.

Nile tilapia (*Oreochromis niloticus*) is an economically important euryhaline fish with high nutritional value and resistance to disease and stress, and can also adapt to a certain level of alkalinity stress [28–30]. Therefore, it is appropriate to use tilapia as the research subject to explore the response and mechanism of aquatic animals to alkalinity stress. In this study, acute alkalinity stress and RNA interference trials were used to determine the importance of proline metabolism in the alkalinity tolerance. The results will provide important clues for understanding the regulatory strategies of tilapia under alkalinity stress, and can serve as theoretical basis for the optimization of saline-alkaline water aquaculture.

## Materials and methods

### Design and synthesis of dsRNA

Various cDNA sequences of the Nile tilapia *pycr* gene obtained from NCBI were aligned to determine its conserved sequence. Specific primers for the double-stranded RNA (*pycr*-dsRNA) were subsequently designed (Table 1). Subsequently, *pycr*-dsRNA was prepared, in short, the target segment was amplified using Nile tilapia liver cDNA as a template according to the instructions (R045B, Takara, Dalian, China), purified, and then ligated with vectors. The resulting plasmids were introduced into recipient cells for cultivation, and bacterial cultures with correct target sequences were selected for sequencing. Plasmids were extracted from correctly sequenced bacteria (9760, Takara, Dalian, China). Following the instructions (R045B, Takara, Dalian, China), target segments containing the T7 promoter (5′TAATACGACTCACTATAGGG3′) were amplified using correctly sequenced plasmids as templates, followed by extracted and in vitro transcription (6140, Takara, Dalian, China). Finally, the concentration and purity were verified via gel electrophoresis (1.2% denaturing agarose) and a Nanodrop 2000, and diethyl pyrocarbonate water was added to adjust the concentration to 5,000 ng/g, and stored at −20 °C for later use.

**Table 1:** Primer pair sequences for *pycr*-dsRNA synthesis and gene primer pair sequences used for real-time PCR (qPCR)

Gene	Position	Primer sequence (5'→3')	Size, bp
*pc*	Forward	ATGTCACACCCGATGCTTCC	20
	Reverse	ATATCGTCTGAACGCCTGCC	20
*pk*	Forward	CAGCATAATCTGCACCATCGGT	22
	Reverse	ATGAGAGAAGTTAAGACGGGCGA	23
*pfk*	Forward	CTGACATGACCATCGGCACT	20
	Reverse	ATCTTCCCCTTCGCAGTCTGT	21
*hk*	Forward	TTCCTCTGGGCTTCACCTTCT	21
	Reverse	ATCTTCCCCTTCGCAGTCTGT	21
*g6pase*	Forward	GGATGCTAATGGGCCTGGTC	20
	Reverse	CAGCTACCAGTGTGCCTGTAA	21
*fbpase*	Forward	ACCGGACAATAGCGGAAAATACA	23
	Reverse	TGGCGAATATTGTTCCTATGGAGA	24
*nhe*	Forward	ATGAAGCGTCAGCCTAGGAA	20
	Reverse	TCCCAGAGCCTGGATCATAC	20
*cftr*	Forward	TCACCAGCATCGCTGTAGATG	21
	Reverse	GGTTGTCGATGACGATATCAGG	22
*nka*	Forward	TTCCCCACTGAGAACTTGTGC	21
	Reverse	ACACCTTTAGCGATGGCCTTG	21
*nkcc*	Forward	ATGGGAGCACTGGATCAGGA	20
	Reverse	TAGGGACCCACTGTAGCGAT	20
*arg1*	Forward	GTGTGATTACCTGTCCGCCA	20
	Reverse	CTGACACAGGTGTTCGGTGA	20
*arg2*	Forward	TCCCCTTCAGGAAACCTCCA	20
	Reverse	TTGGTACTCCCCAGGGTCAA	20
*p5cs*	Forward	AGCCAAGGGCATTCCTGTTT	20
	Reverse	CAATGCTGGCATCACTGTCG	20
*oat*	Forward	GTCCCATTCAACGACATA	18
	Reverse	TCAGCAATCCACAACACA	18
*pycr3*	Forward	TGTCCTTCCTGCAAACGTCA	20
	Reverse	CAAAGACCACATCGGACCCA	20
*pycr1a*	Forward	GTTATGGAGAGCGGTGGCTT	20
	Reverse	ATGGCAGCAGGGGAAATCTT	20
*pycr1b*	Forward	TGATCGCCACACACAGGATT	20
	Reverse	TTCCTCAGTCCCGATACCGT	20
*prodh*	Forward	ACAGAAGAAGAAGAGAGGC	19
	Reverse	GGTAGGTGTTGAAAATGAC	19
*p5cdh*	Forward	AGTCGTTCGGGCGGATAAAG	20
	Reverse	TTCCTCCGGCGATGACTTTC	20
*pycr-*dsrna	Forward	TAATACGACTCACTATAGGGAACCTCACCTGGTTCCGCTA	19
	Reverse	TAATACGACTCACTATAGGGCCACAGCACTCATGGTTGAC	21
*β-actin*	Forward	GGATTCACTCTGAGCGCCG	19
	Reverse	CCGTCTCCTTACCTTTGGGTG	21

*Pc* Pyruvate carboxylase, *pk* Pyruvate kinase, *pfk* Phosphofructokinase, *hk* Hexokinase, *g6pase* Glucose-6-phosphatase, *fbpase* Fructose-1,6-bisphosphatase, *nhe* Na^+^/H^+^ exchanger, *cftr* Cystic fibrosis transmembrane conductance, *nka* Na^+^/K^+^-ATPase, *nkcc* Na^+^-K^+^-2Cl^−^ cotransporter, *arg1* Arginase-1, *arg2* Arginase-2, *p5cs* Δ1-Pyrroline-5-carboxylate synthetase, *oat* Ornithine aminotransferase, *pycr3* Pyrroline-5-carboxylate reductase-3, *pycr1a* Pyrroline-5-carboxylate reductase-1a, *pycr1b* Pyrroline-5-carboxylate reductase-1b, *prodh* Proline dehydrogenase, *p5cdh* Δ1-Pyrroline-5-carboxylate dehydrogenase, the T7 promoter sequences are underlined

### Experimental animals and experimental design

The fish used in this study were procured from Guangdong Tianfa Fish Fry Development Co. Ltd. (China). Before the test, tilapia underwent a 2-week acclimation period within a 500-L tank at the Biological Experimental Station of East China Normal University. During the acclimation period, the fish were fed commercial feed (China Tongwei Group Co., Ltd.) twice daily (at 8:00 and 17:00) until satiety was evident. After 2 weeks of acclimatization, 360 juvenile Nile tilapia (3.61 ± 0.02 g) with similar physical fitness were selected and randomly allocated to twelve 200-L aquaculture tanks with 30 fish in each tank. The 12 fish tanks were randomly divided into 4 groups (FW group, fish cultured in fresh water; AW-2 group, fish cultured in alkaline water with an alkalinity of 23.81 mmol/L; AW-3 group, fish cultured in alkaline water with an alkalinity of 35.71 mmol/L; AW-4 group, fish cultured in alkaline water with an alkalinity of 47.61 mmol/L) for the experiment, with 3 replicates per group. The remaining fish continued to be temporarily cultured. During the stress period, commercial diet was fed with apparent satiety twice a day. At the end of the 2-week stress experiment, the tilapia in each tank were counted to assess the survival rate (SR).

After the acute stress experiment, a total of 180 Nile tilapia, which were healthy and no body surface damage with similar body weight, were selected from the remaining temporary fish and randomly divided into 6 groups (FWb group, fish cultured in fresh water without injection; FWc group, fish cultured in fresh water and injected with sterile saline; FWp group, fish cultured in fresh water and injected with 5,000 ng/g sterile *pycr*-dsRNA; AW group, fish cultured in alkaline water with an alkalinity of 59.52 mmol/L without injection; AWc group, fish cultured in alkaline water with an alkalinity of 59.52 mmol/L and injected with sterile saline; AWp group, fish cultured in alkaline water with an alkalinity of 59.52 mmol/L and injected with 5,000 ng/g sterile *pycr*-dsRNA). There were 3 replicates per group and 10 fish per replicate. The injection site was below the left pectoral fin on the abdominal cavity of each fish. 24 h after injection, fish were exposed to fresh water or 59.52 mmol/L carbonate alkaline water for 48 h, during which death was observed and recorded.

After the survival rate experiment, the above procedure was repeated, and 180 fish were randomly selected from the remaining temporary fish and injected, but 24 h after injection, fish were exposed to fresh water or 59.52 mmol/L carbonate alkaline water for 3 h before sampling. During all trial, the controlled environmental conditions were a temperature of 26 ± 1 °C, a dissolved oxygen concentration ranging from 5.0 to 6.0 mg/L, 12 h of light and 12 h of darkness. In addition, during domestication and 2 weeks of stress, two-thirds of the water in each tank was replaced daily.

### Sample collection

After 2 weeks of acute alkalinity stress, fish were anesthetized with tricaine methanesulfonate (MS-222), then weighed, measured, and counted to determine growth performance. Six fish were randomly selected, and the tail vein blood was rapidly drawn with a 1-mL sterile syringe, then centrifuged at 3,500 r/min for 10 min, and the serum was separated and stored in the refrigerator at −80 °C for subsequent detection. Similarly, six fish were randomly selected, dissected on ice, the liver and visceral masses were weighed, and the liver, gill, and kidney were subsequently collected and immediately stored at −80 °C.

After 3 h of alkalinity stress, 6 fish in each group were randomly selected to be anesthetized on ice, and the blood from tail vein was quickly extracted, then the serum was separated and stored in the refrigerator at −80 °C until tested. The liver, gill and kidney were collected after dissection with sterilized scissors and tweezers and immediately stored at −80 °C. Growth performance parameters were calculated as follows:

$$\begin{array}{l}\mathrm{Weight}\;\mathrm{gain}\;(\mathrm{WG},\%)=100\times(\mathrm{final}\;\mathrm{body}\;\mathrm{weight}\;-\;\mathrm{initial}\;\mathrm{body}\;\mathrm{weight})/\mathrm{initial}\;\mathrm{body}\;\mathrm{weight};\\\mathrm{Survival}\;\mathrm{rate}\;(\mathrm{SR},\;\%)=100\;\times\;(\mathrm{final}\;\mathrm{fish}\;\mathrm{number}/\mathrm{initial}\;\mathrm{fish}\;\mathrm{number});\\\mathrm{Condition}\;\mathrm{factor}\;(\mathrm{CF},\;\%)\;=\;100\times(\mathrm{wet}\;\mathrm{body}\;\mathrm{weight},\;\mathrm g)/{(\mathrm{body}\;\mathrm{length},\;\mathrm{cm})}^3;\\\mathrm{Hepatosomatic}\;\mathrm{index}\;(\mathrm{HSI},\;\%)\;=\;100\times\mathrm{wet}\;\mathrm{hepatopancreas}\;\mathrm{weight}/\mathrm{wet}\;\mathrm{body}\;\mathrm{weight};\\\mathrm{Visceral}\;\mathrm{index}\;(\mathrm{VSI},\;\%)=100\times\mathrm{wet}\;\mathrm{visceral}\;\mathrm{weight}/\mathrm{wet}\;\mathrm{body}\;\mathrm{weight};\\\mathrm{Feed}\;\mathrm{intake}\;(\%)=100\times\mathrm{total}\;\mathrm{feed}\;\mathrm{intake}/\left[\left(\mathrm{final}\;\mathrm{fish}\;\mathrm{number}\;+\;\mathrm{initial}\;\mathrm{fish}\;\mathrm{number}\right)/2\right].\end{array}$$

### Histological analysis

After 3 h of alkalinity stress, livers of three fish in each group were randomly selected, fixed in 4% paraformaldehyde solution for 48 h, washed in 70% ethanol solution, and then transferred to 70% ethanol solution for storage until histological analysis. The subsequent processes were carried out according to the methods in previous articles from our laboratory [31].

### Analysis of ion regulation and ammonia metabolism related indexes

The contents of Na^+^ (C002-1-1), K^+^ (C001-2-1), Cl^−^ (C003-2-1), blood ammonia (A086-1-1) and urea nitrogen (C013-2-1) in serum were determined with commercial kits purchased from Nanjing Jiancheng Bioengineering Institute. The collected gill tissue was homogenized (1:9, w/v) in 0.86% NaCl cold solution, and the homogenate was used to detect the activity of Na^+^/K^+^-ATPase (NKA) and the total protein concentration (A047-1-1, A045-4-2).

### Determination of proline content

According to the manufacturer’s instructions, the content of proline in different tissues (liver, gill, kidney and serum) was quantified by the commercial kit (BC0295, Solarbio Technology Co., Ltd., Beijing, China). The absorbance at a wavelength of 520 nm was measured, and then the proline content was calculated.

### Assay of oxidative stress and antioxidant parameters

The collected liver tissue was homogenized with the appropriate homogenization medium according to the manufacturer's instructions, and the homogenate was separated by centrifugation and stored at −80 °C for later use. The liver homogenate was used to detect the malonaldehyde (MDA, A003-1-2), glutathione (GSH, A061-1-2), glutathione oxidized (GSSG, A061-1-2), triphosphopyridine nucleotide (NADPH, A115-1-1) and nicotinamide adenine dinucleotide phosphate (NADP^+^, A115-1-1) content; total antioxidant capacity (T-AOC, A015-2-1), superoxide dismutase (SOD, A001-3-2), catalase (CAT, A007-1-1), glutathione reductase (GR, A062-1-1) and glutathione peroxidase (GSH-Px, A005-1-2) activities with the kits (Nanjing Jiancheng Bioengineering Institute, Nanjing, China). The total protein concentration (A045-4-2) in liver tissue sample was also determined according to the corresponding commercial kits.

Prior to measuring reactive oxygen species (ROS), the liver cells were isolated. The fish were anesthetized using MS-222, followed by careful extraction of liver samples using sterile scissors and tweezers. The samples were then rinsed in phosphate buffer saline (PBS) solution. After absorption of the PBS, pancreatic enzymes (Shanghai XP Biomed Ltd., China) were added to the liver sample, which was subsequently cut into small pieces (approximately 1 mm^3^) using sterile scissors. These pieces were transferred to a 15-mL sterile centrifuge tube and immersed in water at 28 °C for 20 min. An equal volume of L15 medium (Cytiva Bio-technology Co., Ltd., Hangzhou, China) was added and centrifuged at 1,000 r/min for 5 min. Following removal of the supernatant, a wash with red cell lysate (Tiangen Biotech Co., Ltd., Beijing, China) was performed. The content of ROS (S0033S) in liver was detected with the kit (Beyotime Biotech Inc., Shanghai, China) according to the instructions.

### Glycometabolism related index analysis

Part of the liver samples were weighed and the contents of liver glycogen (A043-1-1) and lactic acid (A019-2-1) were detected with the kits (Nanjing Jiancheng Bioengineering Institute, Nanjing, China) according to the instructions, and the contents of glycogen (A043-1-1) and lactic acid (A019-2-1) in the serum were quantified. The content of adenosine triphosphate (ATP, S0026) in the liver was detected using the corresponding commercial kits (Beyotime Biotech Inc., Shanghai, China), and glucose-6-phosphate dehydrogenase (G6PDH) activity in liver was determined by the commercial kit (BC0265, Solarbio Technology Co., Ltd., Beijing, China).

### Quantitative real-time PCR

Total mRNA from the liver, gill and kidney was extracted using TRIzol reagent (Takara, Dalian, China). The quality and concentration of total RNA were measured by the Nanodrop 2000 spectrophotometer (Thermo Fisher Scientific, Wilmington, USA). Then using the reverse transcription kit (RR047, Takara, Japan) synthesized cDNA and stored at −80 °C. All of the real-time PCR analyses were used an RT‒PCR kit (Vazyme Biotech Co., Ltd., Nanjing, China), and the total reaction volume was 10 μL. The primer sequences of each gene were listed in Table 1, and β-actin was used as reference gene to normalize cDNA loading. The expression of related genes was calculated by the 2^−ΔΔCt^ method.

### Data analysis

SPSS Statistics 27 was used for all statistical analyses. All data are expressed as the mean ± standard error of the mean (SEM) and met the normal distribution and variance homogeneity test. One-way ANOVA of variance was used to analyze the data of the groups treated 2 weeks of alkalinity stress. Then, the independent-samples *t*-test was adopted to analyze data of the groups treated 3 h of alkalinity stress. For all the data, *P* < 0.05 indicated statistical significance. In addition, Pearson correlation analysis was used to analyze the correlation between feed intake and growth performance parameters (WG, CF and SR). Asterisk (*) represents a significant difference between groups (*P* < 0.05). Double star (**) represents a significant difference between groups (*P* < 0.01). Three stars (***) represents a significant difference between groups (*P* < 0.001). A, B, C and a, b, c on bars without a common superscript letter are significantly different (*P* < 0.05) (A/a represents the lowest value).

## Results

### Growth performance and physiological parameters of Nile tilapia after 2 weeks of alkalinity stress

The growth and physiological parameters of tilapia in each group are shown in Table 2. Fish in the fresh water group had the highest rate of WG and CF, while the HSI is lowest (*P* < 0.05). There were no significant differences in VSI among the alkalinity treatment groups (*P* > 0.05). Alkalinity exerted a notable influence on survival rate and feed intake, and the higher the alkalinity, the lower the survival rate and feed intake during the same period (*P* < 0.05). In addition, feed intake was positively correlated with growth performance (WG, CF and SR, *P* < 0.05).

**Table 2:** Growth performance and physiological parameters of *O. niloticus* under acute stress at different alkalinity levels

Diets	Initial weight, g	Final weight, g	WG, %	CF, %	HSI, %	VSI, %	SR, %	Feed intake, %
FW	3.67 ± 0.33	8.41 ± 0.17^c^	130.76 ± 4.54^c^	3.30 ± 0.03^c^	3.09 ± 0.07^a^	12.35 ± 0.19	87.78 ± 1.11^b^	3.72 ± 0.07^c^
AW-2	3.57 ± 0.33	5.65 ± 0.14^b^	57.21 ± 3.90^b^	3.20 ± 0.02^b^	3.57 ± 0.09^b^	12.27 ± 0.14	77.78 ± 2.94^a^	2.79 ± 0.05^b^
AW-3	3.62 ± 0.39	4.77 ± 0.09^a^	32.15 ± 2.38^a^	3.00 ± 0.04^a^	3.52 ± 0.12^b^	12.16 ± 0.14	76.67 ± 1.92^a^	2.51 ± 0.07^a^
AW-4	3.61 ± 0.01	4.72 ± 0.11^a^	30.83 ± 3.16^a^	3.02 ± 0.04^a^	3.75 ± 0.09^b^	12.03 ± 0.12	74.44 ± 2.22^a^	2.44 ± 0.02^a^

Data were expressed as mean ± SEM (standard error of the mean) (*n* = 3 replicate tanks)

^a–c^Values in the same line with different superscripts are significantly different (*P* < 0.05)

Weight gain (WG, %) = 100 × (final body weight − initial body weight)/initial body weight;

Survival rate (SR, %) = 100 × (final fish number/initial fish number);

Condition factor (CF, %) = 100 × (wet body weight, g)/(body length, cm)^3^;

Hepatosomatic index (HSI, %) = 100 × wet hepatopancreas weight/wet body weight;

Visceral index (VSI, %) = 100 × wet visceral weight/wet body weight;

Feed intake (%) = 100 × total feed intake/[(final fish number + initial fish number)/2]

### Proline content and proline metabolism of Nile tilapia after 2 weeks of alkalinity stress

**Fig. 1:**
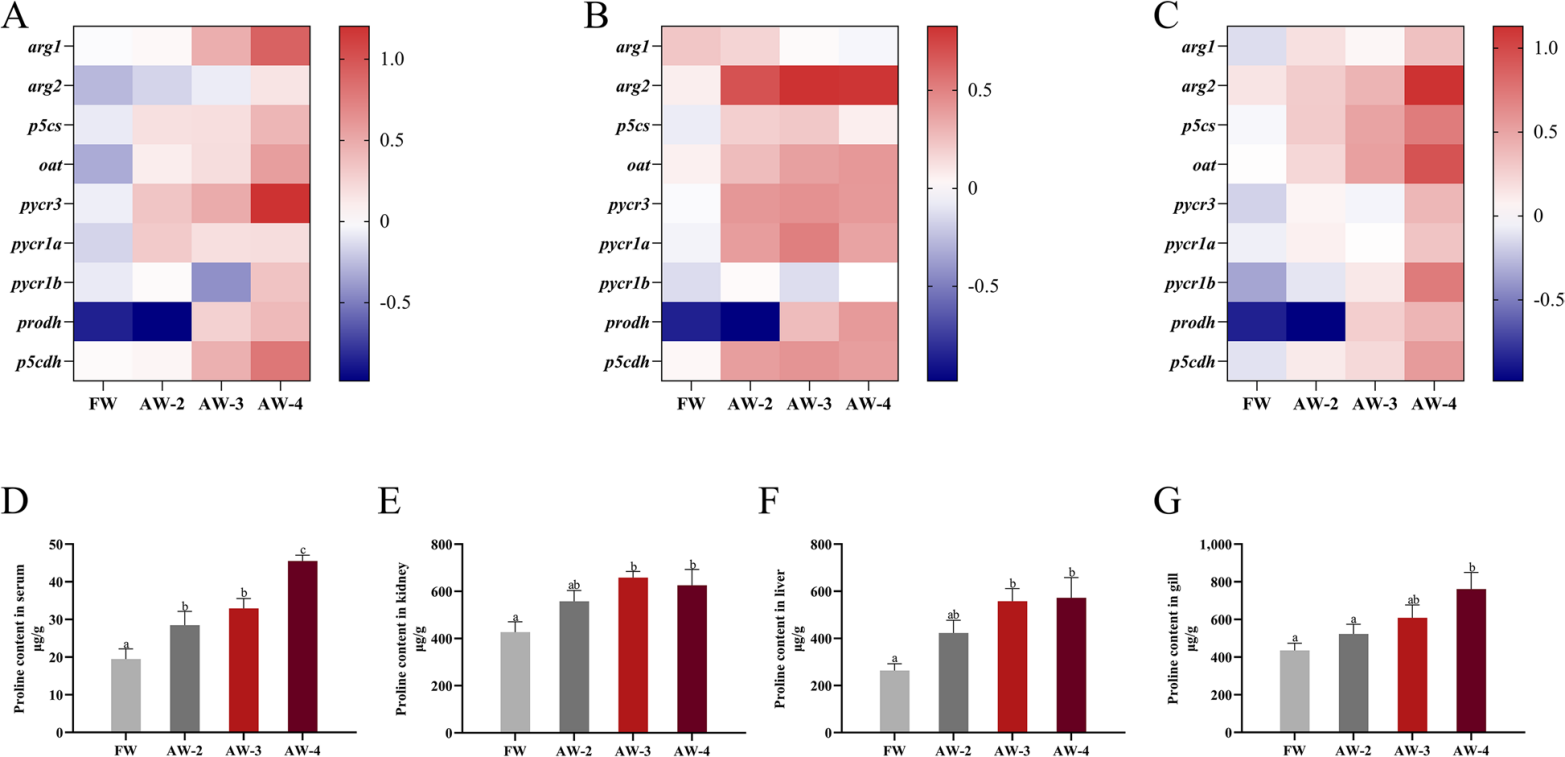
Proline content and q-PCR analysis of proline metabolism-related gene expression of *O. niloticus* under 2 weeks stress at different alkalinity levels. **A** Heatmap showing the mRNA levels of proline metabolism-related genes in liver, with *P* < 0.05; **B** Heatmap showing the mRNA levels of proline metabolism-related genes in gill, with *P* < 0.05; **C** Heatmap showing the mRNA levels of proline metabolism-related genes in kidney, with *P* < 0.05; **D** Proline content in serum; **E** Proline content in kidney; **F** Proline content in liver; **G** Proline content in gill. ^a–c^Bars without a common letter are significantly different (*P* < 0.05)

The contents of proline in liver, gill, kidney and serum of tilapia were affected by alkalinity, and showed a significant upward trend with the increase of alkalinity (*P* < 0.05, Fig. 1D–G). At the same time, alkalinity had significant effects on the mRNA expression of genes related to proline decomposition and synthesis in liver, gill and kidney of tilapia (*P* < 0.05, Fig. 1A–C). In general, the increase of alkalinity in water led to an increase in proline content and a more active proline metabolism of tilapia.

### Interference efficiency of *pycr*-dsRNA

**Fig. 2:**
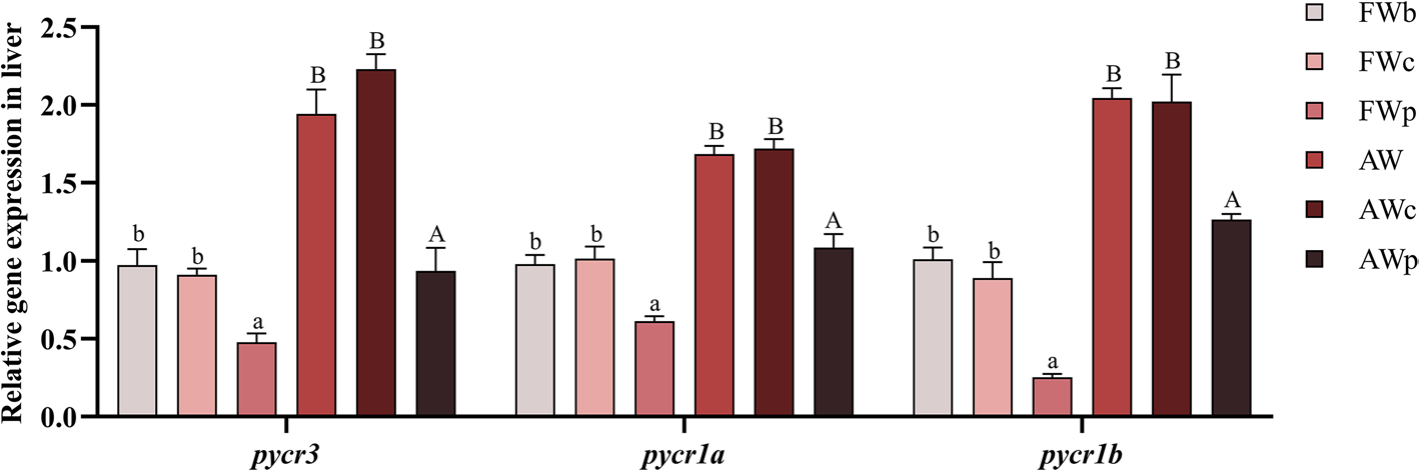
Interference efficiency of *pycr*-dsRNA injection on *pycr* gene mRNA levels of *O. niloticus. *^a,b^Bars without a common letter are significantly different between fresh water groups (*P* < 0.05). ^A,B^Bars without a common letter are significantly different between alkaline water groups (*P* < 0.05)

The interference efficiency of *pycr*-dsRNA is shown in Fig. 2. Compared with the FWc group, the interference efficiency of *pycr*-dsRNA injection on *pycr3*, *pycr1a* and *pycr1b* in the FWp group was 47%, 40% and 72%, respectively. In addition, there was no significant difference in *pycr* mRNA expression between FWb group and FWc group, nor between AW group and AWc group. Therefore, in order to exclude the influence of injection operation itself and facilitate the analysis and discussion of the results, only the FWc group, AWc group and AWp group were selected for analysis and comparison.

### Effect of *pycr*-dsRNA on alkalinity tolerance of Nile tilapia

**Fig. 3:**
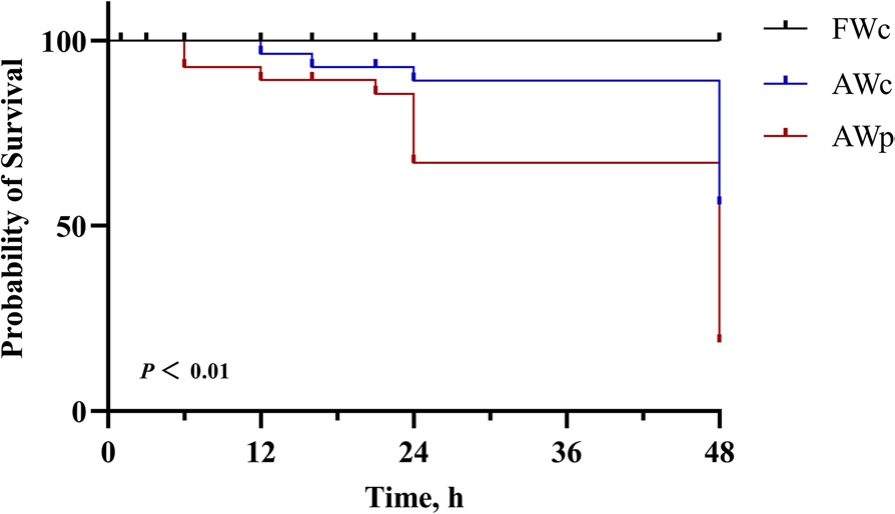
Effect of *pycr*-dsRNA injection on alkalinity tolerance of *O. niloticus*

The survival curve of tilapia after 48 h of alkalinity stress (Fig. 3) shows a survival rate of less than 30% for the AWp group, while the rate for the AWc group was over 50%. FWc, AWc and AWp groups had survival rate of 100%, 58.1% and 26.7%, respectively. In general, the decrease of *pycr* gene mRNA expression significantly reduced the survival rate of tilapia in alkaline water.

### Effect of *pycr*-dsRNA on hepatic oxidative stress related parameters of Nile tilapia

**Fig. 4:**
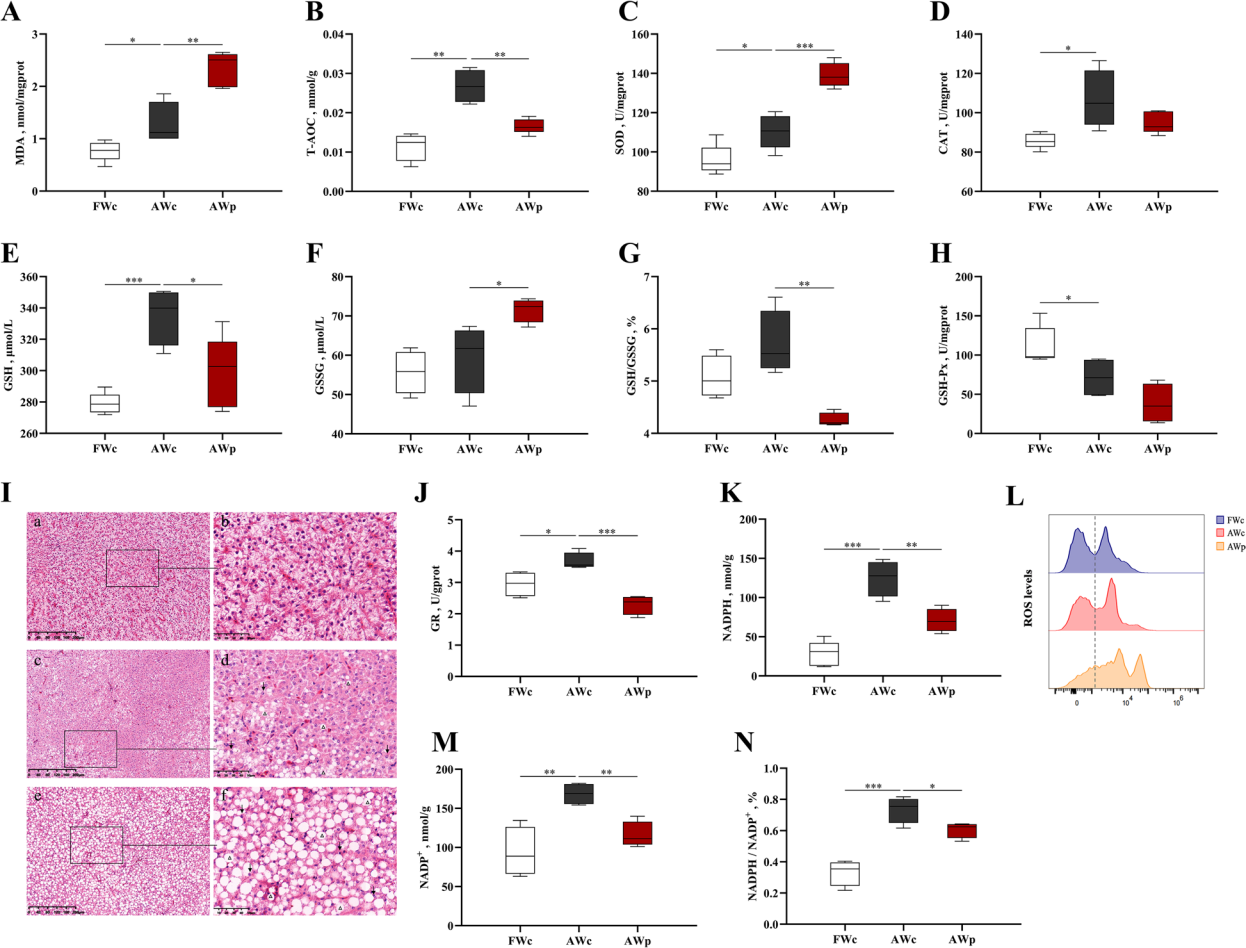
Hepatic oxidative stress analysis of *O. niloticus* after 3 h alkalinity stress after *pycr*-dsRNA injection. **A** MDA content in liver; **B** T-AOC of liver; **C** Activity of SOD in liver; **D** Activity of CAT in liver; **E** GSH content in liver; **F** GSSG content in liver; **G** GSH/GSSG ratio in liver; **H** Activity of GSH-Px in liver; **I** Histopathological changes in the livers of *O. niloticus* in the three groups; **J** Activity of GR in liver; **K** NADPH content in liver; **L** ROS content in liver; **M** NADP^+^ content in liver; **N** NADPH/NADP^+^ ratio in liver. a and b, FWc group; c and d, AWc group; e and f, AWP group. a, c and e, scale bar = 200 μm; b, d and f, scale bar = 50 μm. Arrow indicate nuclei shifted to the periphery of the hepatocytes; Triangle indicate vacuolation. ^*^P < 0.05, ^**^P < 0.01, ^***^P < 0.001

After alkalinity stress, MDA content in liver was significantly increased, as were the activities of T-AOC, SOD and CAT. The MDA content in liver of Nile tilapia was further increased after injection of *pycr*-dsRNA, while the activity of T-AOC was significantly decreased (*P* < 0.05, Fig. 4A–D). In addition, alkalinity stress caused a decrease in the activity of GSH-Px in liver, and an increase in the activity of GR and the content of GSH. After injection of *pycr*-dsRNA, the activity of GR and the GSH content were significantly decreased, the GSSG content accumulated, and the body redox balance was destroyed (*P* < 0.05, Fig. 4E–H and J). Meanwhile, alkalinity stress caused a significant increase in total NADPH and total NADP^+^ in the liver, and NADPH/NADP^+^ ratio decreased significantly after the mRNA expression of *pycr* decreased (*P* < 0.05, Fig. 4K, M and N).

The liver morphology of fish in the FWc group was normal with no obvious abnormalities, while there were obvious vacuoles in liver cytoplasm in the AWc group, and the number of vacuoles in the hepatocyte cytoplasm significantly increased after *pycr*-dsRNA injection (*P* < 0.05, Fig. 4I). Alkalinity stress caused ROS accumulation in the liver, and the ROS content was significantly increased after *pycr*-dsRNA injection (*P* < 0.05, Fig. 4L).

### Effects of *pycr*-dsRNA on serum ammonia content, plasma urea nitrogen content and ion regulation of Nile tilapia

**Fig. 5:**
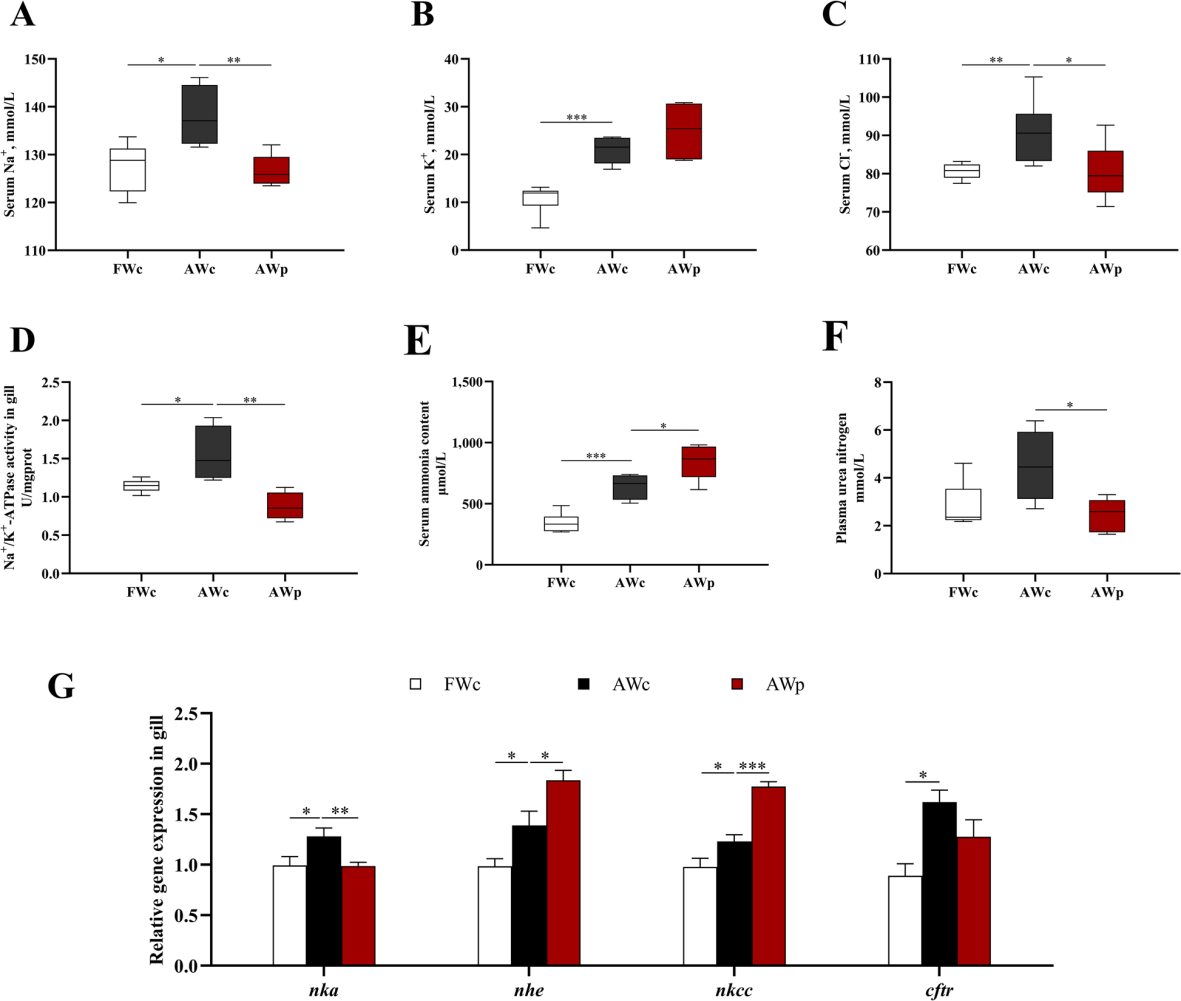
Serum ammonia content, plasma urea nitrogen content, serum ion content, NKA activity and q-PCR analysis of gill ion transporter gene expression of *O. niloticus*in after 3 h alkalinity stress after *pycr*-dsRNA injection. **A** Serum Na^+^ content; **B** Serum K^+^ content; **C** Serum Cl^−^ content; **D** Activity of NKA in gill; **E** Serum ammonia content; **F** Plasma urea nitrogen content; **G** Expression of *nka*, *nhe*, *nkcc* and *cftr* genes in gill. ^*^P < 0.05, ^**^P < 0.01, ^***^P < 0.001

The contents of Na^+^, K^+^ and Cl^−^ in serum were significantly increased due to alkalinity stress, and the contents of Na^+^ and Cl^−^ in serum were significantly decreased after *pycr*-dsRNA injection (*P* < 0.05, Fig. 5A–C). The mRNA expression of *nka*, Na^+^/H^+^ exchanger (*nhe*), cystic fibrosis transmembrane conductance (*cftr*) and Na^+^-K^+^-2Cl^−^ cotransporter (*nkcc*) and NKA activity in the gill were significantly increased under alkalinity stress. After *pycr*-dsRNA injection, the mRNA expression and enzyme activity of NKA were significantly decreased, while the mRNA expressions of *nhe* and *nkcc* were significantly increased (*P* < 0.05, Fig. 5D and G). In addition, after alkalinity stress, the serum ammonia content was significantly increased, and after injection of *pycr*-dsRNA, the serum ammonia content was further increased, while the plasma urea nitrogen content was significantly decreased (*P* < 0.05, Fig. 5E and F).

### Effects of *pycr*-dsRNA on glycometabolism of Nile tilapia

**Fig. 6:**
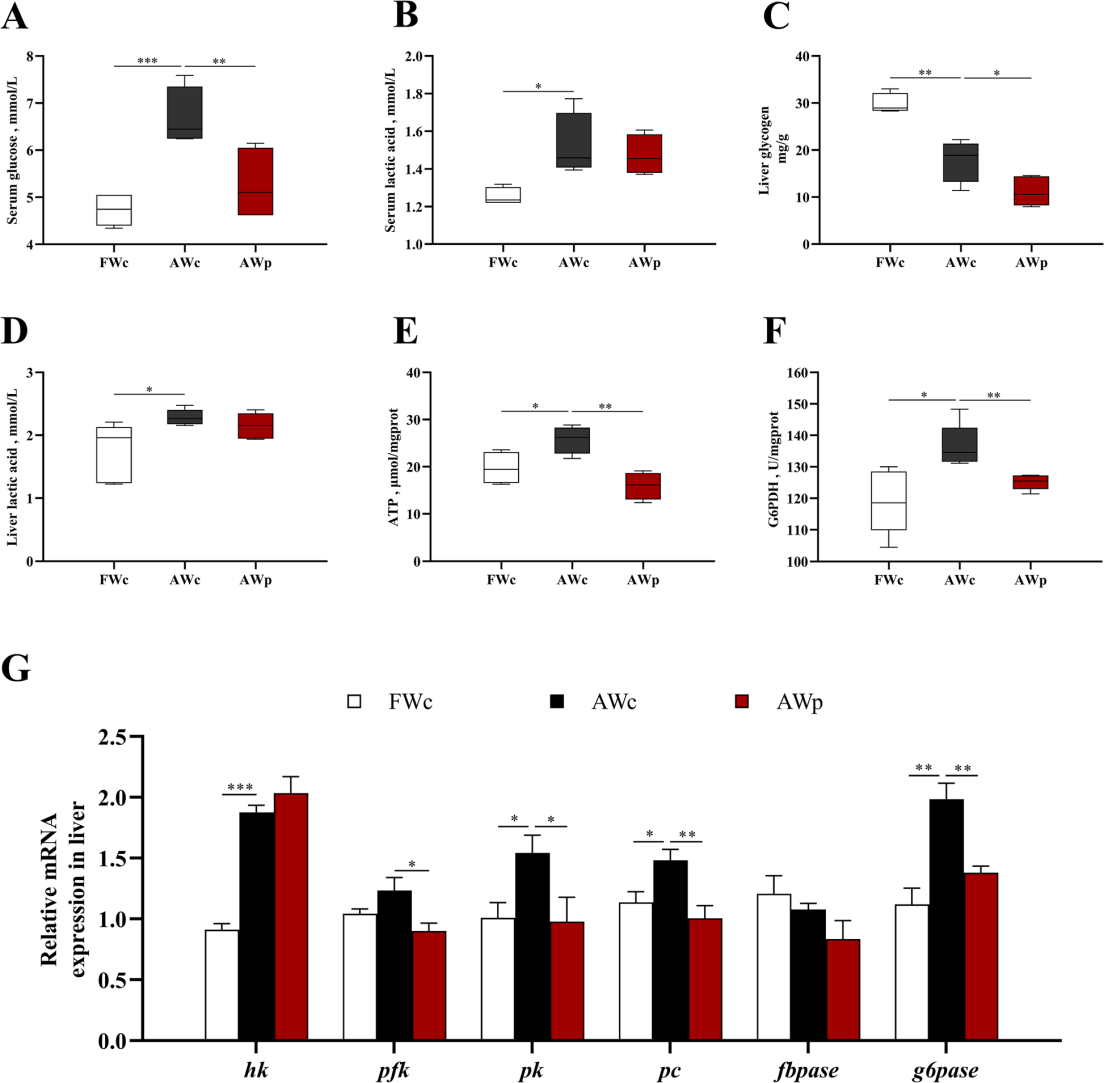
Glycometabolism analysis of *O. niloticus*in after 3 h alkalinity stress after *pycr*-dsRNA injection. **A** Serum glucose content; **B** Serum lactic acid content; **C** Liver glycogen content; **D** Liver lactic acid content; **E** ATP content in liver; **F** Activity of G6PDH in liver; **G** Expression of *hk*, *pfk*, *pk, pc, fbpase* and *g6pase* genes in liver. ^*^P < 0.05, ^**^P < 0.01, ^***^P < 0.001

After alkalinity stress, the contents of serum glucose, lactic acid and liver lactic acid and ATP were significantly increased, while the contents of liver glycogen were significantly decreased. After injection of *pycr*-dsRNA, the contents of serum glucose, liver ATP and liver glycogen were significantly decreased (*P* < 0.05, Fig. 6A–E). At the same time, the mRNA expressions of hexokinase (*hk*), pyruvate kinase (*pk*), pyruvate carboxylase (*pc*), glucose-6-phosphatase (*g6pase*) and the activity of G6PDH in liver were significantly increased, while the mRNA expressions of phosphofructokinase (*pfk*), *pc*, *g6pase* and the activity of G6PDH were significantly decreased after injection of *pycr*-dsRNA (*P* < 0.05, Fig. 6F and G).

### Effects of *pycr*-dsRNA on proline content and proline metabolism of Nile tilapia

**Fig. 7:**
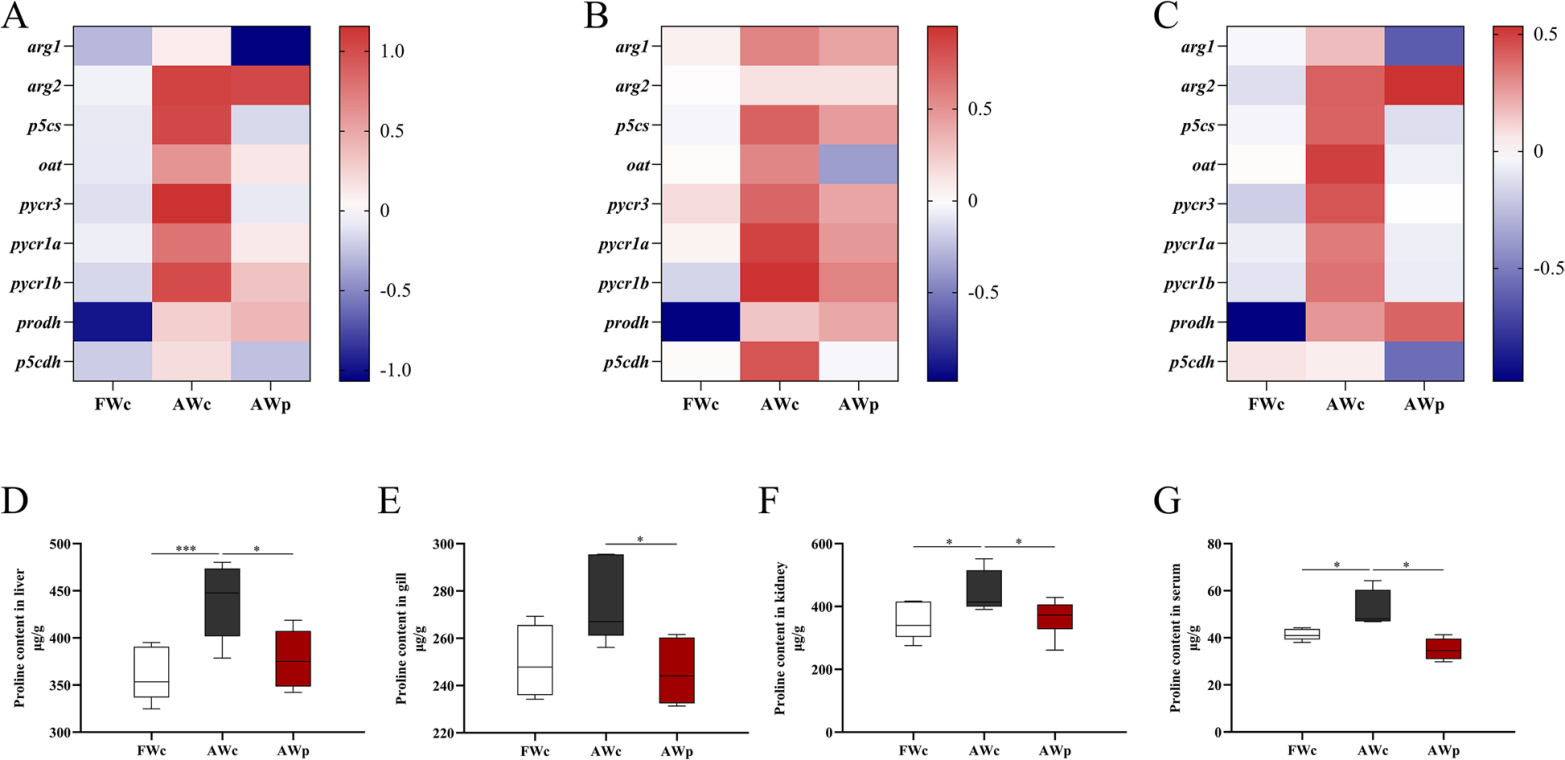
Proline content and q-PCR analysis of proline metabolism-related gene expression of *O. niloticus*in after 3 h alkalinity stress after *pycr*-dsRNA injection. **A** Heatmap showing the mRNA levels of proline metabolism-related genes in liver, with *P* < 0.05; **B** Heatmap showing the mRNA levels of proline metabolism-related genes in gill, with *P* < 0.05; **C** Heatmap showing the mRNA levels of proline metabolism-related genes in kidney, with *P* < 0.05; **D** Proline content in liver; **E** Proline content in gill; **F** Proline content in kidney; **G** Proline content in serum. ^*^P < 0.05, ^***^P < 0.001

The contents of proline in liver, kidney and serum were significantly increased after alkalinity stress, while the contents of proline in liver, gill, kidney and serum were significantly decreased after *pycr*-dsRNA injection (*P* < 0.05, Fig. 7D–G). Meanwhile, the mRNA expressions of genes related to proline decomposition and synthesis in liver, gill and kidney were significantly increased under alkalinity stress, but decreased significantly after *pycr*-dsRNA injection (*P* < 0.05, Fig. 7A–C).

## Discussion

Alkalinity has always been considered as a non-biological hazard factor for the survival of aquatic animals [32]. Previous studies have shown that alkalinity can cause chronic and acute damage to aquatic organisms, posing a threat to their growth and survival [9, 33–35]. The results of this study showed that tilapia in alkaline water exhibit lower WG, CF and SR compared to those in fresh water, and these parameters decreased further with increasing alkalinity. Organisms consumed more energy to maintain physiological homeostasis in alkaline water, which resulted in less energy for growth and lower survival rate under alkalinity stress [36]. This is consistent with previous studies on other fish, such as Songpu mirror carp (*Cyprinus carpio Songpua*) and Shemaia (*Chalcalburnus chalcoides aralensis*) [37, 38]. Additionally, this study demonstrates a significant positive correlation between feed intake and growth performance, suggesting that an important factor in the reduction of growth performance of organisms caused by alkalinity stress is reduced feed intake. Therefore, adjusting feeding strategies or using attractants to increase feed intake may enhance the production value and economic benefits of aquaculture fish within the tolerance range of alkalinity stress.

The effect of carbonate alkalinity stress on aquatic organisms involves several aspects, among which the alteration of amino acid metabolism is a main one [33, 39]. Research on crucian carp (*Carassius auratus*) have showed that under alkalinity stress, various metabolic pathways including glycine, serine and threonine were changed [40]. Similarly, the total plasma free amino acid content of Amur ide (*Leuciscus waleckii*) increased with the increase of alkalinity and exposure time, among which the highest changes were proline and valine [41]. This study showed that both the synthesis and degradation of proline in tilapia are enhanced under carbonate alkalinity stress. This is similar with the results of studies on some animals under osmotic stress conditions, such as river nerite (*Theodoxus fluviatilis*), harpacticoid copepod (*Tigriopus brevicornis*) and blue crab (*Callinectes sapidus*) [42–45]. The upregulation of proline synthesis can help utilize the properties of amino acids themselves, such as osmoprotection, radical scavenging, and alleviation of endoplasmic reticulum stress, on the flip side, the upregulation of proline degradation can increase the availability of proline as stress substrate, including for energy supply and signal pathway modulation [46–48]. However, when *pycr* gene was knocked down, both proline synthesis and catabolism were decreased. Meanwhile, the survival rate of tilapia in alkaline water was further decreased. Therefore, proline metabolism has the potential to be a noteworthy response pathway under alkalinity stress.

Alkalinity stress has been reported to disrupt the homeostasis between the oxidative and antioxidant systems of aquatic organisms, inducing the production of ROS and the accumulation of MDA in vivo, while the prolonged presence of ROS can lead to oxidative stress and liver damage, and MDA reflects the degree of oxidative damage [36, 49, 50]. T-AOC is the total antioxidant capacity of the body, including total enzymes and non-enzymes, and its level reflects the antioxidant capacity of the body [51]. In this study, the activity of T-AOC and the contents of ROS and MDA in the liver were significantly increased by alkalinity stress. Meanwhile, alkalinity caused the appearance of liver cytoplasmic vacuoles, indicating liver damage caused by the disrupted balance of ROS production and clearance in the antioxidant system. Under stress, SOD and CAT, which can convert superoxide anion radical to H_2_O_2_ and further reduce H_2_O_2_ to O_2_ and H_2_O, is generally considered as the primary defense line against ROS [52]. In addition to SOD and CAT, GSH-Px can neutralize H_2_O_2_ [53]. In this study, the activity of SOD and CAT increased under alkalinity stress, while the activity of GSH-Px was inhibited. Similar findings have been found in gibel carp (*Carassius auratus gibelio*) and Amur minnow (*Phoxinus lagowskii*) [54, 55]. GSH, a crucial non-enzymatic antioxidant, can clear ROS and thus be oxidized to GSSG [56]. In addition, GR and GSH-Px ensure the reduced glutathione pool by coupling with NADPH/NADP^+^ ratio [57, 58]. We found that alkalinity stress resulted in the increase of GSH content and GR activity, along with elevated levels of NADPH, NADP^+^ and NADPH/NADP^+^ ratio, indicating the activation of the antioxidant network to maintain redox homeostasis under alkalinity stress. However, the inhibition of *pycr* caused further vacuolar degeneration, cell enlargement, and nuclear displacement in liver tissues, which further aggravated oxidative damage of fish liver. Furthermore, GSSG content increased significantly, while GSH, GSH/GSSG ratio, NADPH, NADP^+^ and NADPH/NADP^+^ ratio decreased significantly. The entire antioxidant defense system of the organism severely damaged. The proline cycle can transfer the reduction potential from cytoplasm to mitochondria and oxidation potential from mitochondria to cytoplasm, which can keep redox homeostasis, including maintaining NADPH/NADP^+^ ratio and providing electrons for antioxidant enzymes and decomposition reactions [57, 59–61]. Failure of GR binding to its cofactor NADPH leads to a failure in GSH regeneration from its oxidised form GSSG during detoxification, ultimately leading to a decrease in glutathione levels, a lower GSH/GSSG ratio [62–64]. Similar to our findings, studies on human fibroblasts suggested that silencing of *pycr* directly leads to mitochondrial fragmentation, rendering cells sensitive to oxidative stress [65]. Research on zebrafish (*Danio rerio*) also showed a significant decrease in antioxidant capacity and mitochondrial dysfunction after inhibition of proline biosynthesis [66]. Therefore, proline metabolism contributes to maintaining redox homeostasis and stability of the antioxidant system in tilapia under alkalinity stress, thus helping the body cope with stress.

Under the synergistic effect of high pH and high concentrations of CO_3_^2−^ and HCO_3_^−^ in carbonate alkalinity, alkalinity stress can also cause the ionic toxicity and acid–base imbalance [67]. The ion homeostasis of tilapia under alkalinity stress depends on multiple ion pumps and transporters, including NKA, NHE, CFTR and NKCC [31]. NKA generates an electrochemical gradient by exchanging Na^+^ and K^+^, which not only provides a channel initial force for other transport systems within the gill epithelial, but also collaborates with NHE to capture NH_3_ into NH_4_^+^ and facilitates the excretion of branchial ammonia [68, 69]. NKCC transports Na^+^, K^+^, and Cl^−^, maintaining intracellular Cl^−^ concentration and regulating cell volume [70]. In this study, the gene expressions of *nka*, *nkcc* and *cftr* of tilapia exposed to alkaline water were higher than those of tilapia in fresh water, indicating that tilapia under alkalinity stress maintains acid–base balance and osmotic balance by activating the ion transport system. However, the inhibition of *pycr* can inhibit the gene expression and enzyme activity of *nka*, and increase the gene expressions of *nhe* and *nkcc*. The possible reason is that amino acids are substrates for energy metabolism under stress conditions in fish, and proline is one of the preferred amino acids. When proline synthesis is inhibited, NKA cannot obtain sufficient energy to participate in cellular ion transport, thereby affecting the entire ion efflux system [25]. Additionally, the imbalance of H^+^ caused by alkalinity stress also disrupts the balance of NH_3_ and NH_4_^+^ mutual conversion, further leading to the accumulation of serum ammonia, which may affect the function and structure of the gill endoplasmic reticulum (ER), a crucial organelle responsible for protein synthesis, processing, transport and calcium storage [71–74]. The accumulation of proline can stabilize protein folding and/or promote protein refolding, thus avoiding and/or alleviating ER stress [46]. Inhibition of proline synthesis leads to elevated blood ammonia, which may result enhanced ER stress, further impairing the synthesis and maturation of ion transport proteins [75]. Meanwhile, the oxidative imbalance caused by the obstruction of proline metabolism promoted the upregulation of *nhe* and *nkcc* expression. In summary, tilapia under alkalinity stress maintain physiological balance by activating the ion transport system and ammonia metabolism. Among these processes, proline metabolism plays a crucial role to help fish adapt to alkalinity stress [69].

In response to environmental stressors, aquatic organisms activate various regulatory processes by adjusting energy metabolism. Among these, carbohydrate is the direct energy source for aquatic organisms to resist environmental stress [76, 77]. Glucose metabolism is an important pathway of carbohydrate metabolism, including glucose utilization (glycolysis) and generation (gluconeogenesis) [78]. Glycolysis is the only way for fish to decomposition glucose, and has been shown to be significantly activated under environmental stress conditions such as hypoxia and chemical pesticides [79, 80]. In our study, tilapia showed elevated levels of serum glucose and lactate, hepatic ATP and lactate, as well as significantly upregulated expression levels of glycolysis-related genes (*hk*, *pk*), which confirmed promoting glycolysis was enhanced in fish under alkalinity stress. Gluconeogenesis is another important pathway that converts non-carbohydrates into glucose to produce ATP [81]. We found that the expressions of *pc* and *g6pase* were significantly upregulated under alkalinity stress. However, when the function of *pycr* gene was inhibited, both the glycolysis and gluconeogenesis were suppressed, resulting in significant decreases in hepatic ATP content and serum glucose content. G6PDH is a rate-limiting enzyme in the pentose phosphate pathway (PPP), which can convert the intracellular carbohydrate decomposition process from glycolysis to PPP, and plays an important biological role in nucleotide and fatty acid synthesis and maintenance of intracellular redox homeostasis [82]. In this study, the activity of G6PDH was increased by alkalinity stress, and decreased after the decrease of *pycr* gene expression. This might be because that the changed proline biogeneration and NADPH/NADP^+^ balance affected PPP. Our data suggest that the effect of the proline metabolic axis on glycolysis and PPP under alkalinity stress may be due to its maintenance of NAD(P)^+^ and NAD(P)H [83]. In addition, PRODH-mediated proline degradation helps maintain ATP levels in cells, while PYCRs are able to maintain PRODH activity in the absence of proline [20, 84]. In order to meet the energy requirements of fish during alkaline stress, glycogen is also mobilized. Our results showed a significant decrease in hepatic glycogen content under alkalinity stress, and a further decrease when *pycr* gene expression is reduced. In summary, tilapia regulates several pathways through proline metabolism to compensate and meet the energy demand under alkalinity stress.

As a euryhaline fish, Nile tilapia has a certain degree of alkalinity tolerance. But alkalinity stress still poses challenge to the health, growth and survival of tilapia. In this study, proline synthesis and degradation were activated in Nile tilapia under alkalinity stress, while knocking down the *pycr* gene significantly affects the normal operation of the proline metabolic axis. Meanwhile, the defunction of *pycr* and abnormal proline metabolism affect the ion transport, antioxidant defense system and energy metabolism, which are supposed to play crucial roles in alkalinity stress. These changes then resulted in liver damage and fish death under alkalinity stress. In summary, proline metabolism plays a crucial role in the alkaline adaptation of Nile tilapia. Further studies are warranted to the mechanism of proline metabolic pathway in response to alkalinity stress.

## Conclusion

In conclusion, our study suggests that the proline metabolic axis can serve as a scaffold for the integration of multiple regulatory mechanisms and play a crucial role in the alkaline adaptation of Nile tilapia, which can not only promote energy supply, ion transport and pentose phosphate pathway in fish, but also promote the stability of the antioxidant system by maintaining NAD(P)^+^ and NAD(P)H. Future studies should delve deeper into the regulation of proline metabolism in order to reveal and improve the regulation and signal network of alkalinity stress response.

## Data Availability

The datasets produced and/or analyzed during the current study are available from the corresponding author on reasonable request.

## Abbreviations

ARG1: Arginase-1
ARG2: Arginase-2
ATP: Adenosine triphosphate
CAT: Catalase
CF: Condition factor
CFTR: Cystic fibrosis transmembrane conductance regulator
ER: Endoplasmic reticulum
ETC: Electron transport chain
FBPASE: Fructose-1,6-bisphosphatase
GR: Glutathione reductase
GSH: Glutathione
GSH-Px: Glutathione peroxidase
GSSG: Glutathione disulfide
G6PASE: Glucose-6-phosphatase
G6PDH: Glucose-6-phosphate dehydrogenase
HK: Hexokinase
HSI: Hepatosomatic index
MDA: Malonaldehyde
MS-222: Tricaine methanesulfonate
NADPH: Triphosphopyridine nucleotide
NADP^+^: Nicotinamide adenine dinucleotide phosphate
NHE: Na^+^/H^+^ exchanger
NKA: Na^+^/K^+^-ATPase
NKCC: Na^+^-K^+^-2Cl^−^ cotransporter
OAT: Ornithine aminotransferase
PBS: Phosphate buffer saline
PC: Pyruvate carboxylase
PFK: Phosphofructokinase
PK: Pyruvate kinase
PPP: Pentose phosphate pathway
PRODH: Proline dehydrogenase
PYCR: Pyrroline-5-carboxylate reductase
PYCR1A: Pyrroline-5-carboxylate reductase-1a
PYCR1B: Pyrroline-5-carboxylate reductase-1b
PYCR3: Pyrroline-5-carboxylate reductase-3
P5CS: Δ1-Pyrroline-5-carboxylate synthetase
P5CDH: Δ1-Pyrroline-5-carboxylate dehydrogenase
ROS: Reactive oxygen species
SOD: Superoxide dismutase
SR: Survival rate
T-AOC: Total antioxidant capacity
TCA: Tricarboxylic acid cycle
VSI: Visceral index
WG: Weight gain
